# ChatGPT or LLMs can provide treatment suggestions for critical patients with antibiotic-resistant infections: a next-generation revolution for medical science?

**DOI:** 10.1097/JS9.0000000000000987

**Published:** 2023-12-12

**Authors:** Chiranjib Chakraborty, Soumen Pal, Manojit Bhattacharya, Md. Aminul Islam

**Affiliations:** a Department of Biotechnology, School of Life Science and Biotechnology, Adamas University, Kolkata, West Bengal, India; b School of Mechanical Engineering, Vellore Institute of Technology, Vellore, Tamil Nadu, India; c Department of Zoology, Fakir Mohan University, Vyasa Vihar, Balasore, Odisha, India; d COVID-19 Diagnostic Lab, Department of Microbiology, Noakhali Science and Technology University, Noakhali; e Advanced Molecular Lab, Department of Microbiology, President Abdul Hamid Medical College, Karimganj, Kishoreganj, Bangladesh

*Dear Editor,*

Recently, the different applications of the generative artificial intelligence (AI)-enabled ChatGPT have been increasing almost every day. In this direction, Wu and Zhang^1^ published an article in this journal about ChatGPT’s applications and challenges in healthcare. Similarly, this journal illustrated ChatGPT’s application in drug discovery and development^2^. Both the articles are very timely. However, it is time to explore the other medical applications of ChatGPT or LLMs (large language models).

Generative AI offers a transformative platform to the users. It denotes that deep learning (DL) models are capable of generating good-quality text dependent on the data using the trained model. Generative AI has thrived in the last few years to generate text and has been used for different purposes. However, This class of machine learning (ML) technologies can develop new content such as image, text, video, or music by exploring patterns from the existing data^3,4^. Recently, generative AI has created great hope for researchers and other stakeholders. After the release of ChatGPT, a generative AI-based chatbot powered by LLM, the users’ hope has magnified. ChatGPT’s different uses have been explored in several areas, especially medical science. ChatGPT is OpenAI’s (San Francisco, USA) successful LLM product, and the present model is a free online version that belongs to the GPT-3.5 series. As reported by Brynjolfsson *et al*., the GPT-3 model trains on 300 billion tokens and includes 175 billion parameters; and therefore the GPT-3 model can yield about $5 million in computing costs alone. Similarly, the GPT-4 model is trained on 13 trillion tokens and includes ~1.8 trillion parameters. It can generate a computing-only cost of $65 million^5^. Further, through reinforcement learning, generative AI models can also learn natural images, several different data types, software code, etc. The applications of this generative-enabled technology tool are growing rapidly, and we are just starting to explore the possibilities. Here, we explore the possibilities of providing treatment suggestions from ChatGPT for critical patients with antibiotic-resistant infections.

Infections caused by antibiotic-resistance bacteria seriously threaten the present healthcare system. Antimicrobial resistance (AMR) is one of the severe concerns to public health. It is rapidly rising not only in developing countries but also in other parts of the world. Different reports indicate that AMR is increasingly becoming dangerous in every part of the world. It is a severe bottleneck for treating common infectious diseases such as foodborne ailments, tuberculosis, pneumonia, UTIs, etc. AMR can lead to severe conditions which result in lengthy hospital admissions and, therefore, increase healthcare costs. It causes a reduction in efficiency in fighting infectious agents. Finally, if multi-antibiotic resistance occurs in the patients, the treatment failures were noted^6,7^. Deaths and disability are common results of infections that occur through antibiotic-resistance bacteria. Scientists are investigating to capture the scenario of the global burden of infections caused by antibiotic-resistance bacteria. In this direction, Cassini *et al*. tried to evaluate deaths and disability caused by infections from antibiotic-resistance bacteria in the European Union. They found that the phenomenon was highest in the population aged 65 years or above, and among infants aged less than 1 year. It was highest in Greece and Italy, which has been augmented since 2007^8^. During the global disease burden calculation, Murray *et al*. predicted that due to the emergence and rise of quick AMR, about 10 million people will die by 2050^9^. In case of critical infection, clinicians need to make decisions for the treatment. However, AI has been helping diagnose and treat infectious diseases. Several researchers have used AI to diagnose, treat, and monitor infectious diseases, which are immensely important in public health. Recently, Theodosiou and Read illustrated that AI is used in clinical imaging analysis for diagnosis, clinical decision support tools such as antimicrobial prescribing, sepsis prediction, etc., and in the management of public health. Therefore, AI-based tools have been used to manage the infection^10^. At the same time, the AI-enabled ChatGPT can provide treatment suggestions for different diseases, including infectious diseases^11,12^. Chakraborty *et al*. have commented that DL research might be beneficial for the diagnosis and treatment of antibiotic resistance, and should be encouraged for the benefit of society. They have shown a direction of how the DL models solve the antibiotic resistance problem with proper diagnosis and treatment^13^. ChatGPT is a DL-based state-of-the-art NLP (natural language processing) model. Howard *et al*. recently showed that the AI-enabled ChatGPT can give antimicrobial advice. They asked ChatGPT questions for antimicrobial assistance in eight hypothetical infection situations. They also noted that the antimicrobial regimens were suitable for treating clinical response. At the same time, they reported that the generic course lengths provided by ChatGPT were correct and appropriate. ChatGPT has the capability to recognize antimicrobial contraindications (AMC). However, the AMC did not correlate with the importance^14^.

Here, we have explored ChatGPT’s ability to provide treatment suggestions for antibiotic-resistance infections and found that ChatGPT can provide these suggestions for critical patients with antibiotic-resistance infections. We have asked ChatGPT two critical questions to obtain suggestions for antibiotic-resistance infections. The first question was related to the treatment suggestions for patients with antibiotic-resistance infection, with the first-line and second-line antibiotic-resistance. Similarly, the second question was about the treatment suggestions for patients with first-line, second-line, and supplemental antibiotic resistance. ChatGPT answers both questions with proper justifications (Annexure 1, Supplemental Digital Content 1, http://links.lww.com/JS9/B569).

The number of remote general practitioners (GPs) prescribing antibiotics, such as through telemedicine, is increasing. Recently, a study in connection to acute respiratory infections evaluated the number of remote GPs versus face-to-face GPs in England who are prescribing antibiotics. In this investigation, researchers found higher rates of antibiotic prescription in remote GPs, and the number of remote GPs is increasing^15^. Therefore, the same model can be implemented using ChatGPT to provide treatment suggestions for critical patients with antibiotic-resistance, infections. ChatGPT might act as a remote GP. However, in that case, generative AI-based ChatGPT or other LLM should provide accurate treatment suggestions.

Enormous promises have been visualized for ChatGPT or LLMs. These generative AI-based technologies are accepted and utilized very fast by users. However, we should be conscious of the threats and challenges while addressing the treatment suggestions for antibiotic-resistance infections. Therefore, we urge all stakeholders to educate themselves about new technologies like generative AI, LLM, or next-generation tailor-made models so that they can utilize and benefit from them.

## Ethical approval

Not applicable.

## Sources of funding

None.

## Author contribution

C.C.: conceptualization, data curation, investigation, writing – original draft, and writing – review and editing; S.P.: validation and editing; M.B. and Md.A.I.: validation. All authors critically reviewed and approved the final version of the manuscript.

## Conflicts of interest disclosure

All authors report no conflicts of interest relevant to this article.

## Research registration unique identifying number (UIN)

1. Name of the registry: not applicable.
2. Unique identifying number or registration ID: not applicable.
3. Hyperlink to your specific registration (must be publicly accessible and will be checked): not applicable.

## Guarantor

Md. Aminul Islam, COVID-19 Diagnostic Lab, Department of Microbiology, Noakhali Science and Technology University, Noakhali 3814, Bangladesh; e-mail: aminulmbg@gmail.com.

## Data availability statement

The data in this correspondence article are not sensitive in nature and are accessible in the public domain. The data are therefore available and not of a confidential nature.

## Provenance and peer review

Not commissioned, internally peer-reviewed.

## Supplementary Material

SUPPLEMENTARY MATERIAL
