# Supplementary material for: ChatGPT or LLMs can provide treatment suggestions for critical patients with antibiotic-resistant infections: a next-generation revolution for medical science?
Source: Int J Surg. 2023 Dec 12;110(3):1829–31. doi: 10.1097/JS9.0000000000000987 (PMC10942188; doi:10.1097/JS9.0000000000000987)
Supplement: SUPPLEMENTARY MATERIAL [file js9-110-1829-s001.docx]

**Annexture-1**

**Question 1:** A patient has the following antibiotic resistance profile of FIRST-LINE and SECOND-LINE ANTIBIOTICS:

FIRST LINE ANTIBIOTICS

GENTAMICIN RESISTANT; TRIMETHOPRIM-SULFAMETHOXAZOLE RESISTANT

SECOND LINE ANTIBIOTICS

AMOXYCILLIN-CLAVULANATE RESISTANT; PIPERACILLIN-TAZOBACTAM RESISTANT; CIPROFLOXACIN RESISTANT; CEFUROXIME RESISTANT; CEFTRIAXONE RESISTANT; CEFOPERAZONE-SULBACTAM RESISTANT; AMIKACIN RESISTANT

Which antibiotic can be used for treatment purposes?

**ChatGPT’s Answer:**

The antibiotic resistance profile you provided indicates resistance to a wide range of both first-line and second-line antibiotics. However, there are still some antibiotics that may be effective for treatment. Based on the information provided, one potential option could be Meropenem, which is a carbapenem antibiotic. Carbapenems are often considered as a last resort for treating infections caused by multidrug-resistant bacteria.

It's important to note that the choice of antibiotic should be made by a healthcare professional based on a thorough evaluation of the patient's condition, the specific bacteria causing the infection, and local resistance patterns. If the patient has a severe infection and a history of antibiotic resistance, consultation with an infectious disease specialist is often recommended to determine the most appropriate treatment strategy.

**Question 2:** A patient has the following antibiotic resistance profile of FIRST-LINE, SECOND-LINE and SUPPLEMENTAL ANTIBIOTICS:

FIRST LINE ANTIBIOTICS

GENTAMICIN RESISTANT; TRIMETHOPRIM-SULFAMETHOXAZOLE RESISTANT

SECOND LINE ANTIBIOTICS

IMIPENEM RESISTANT; ERTAPENEM RESISTANT; CEFUROXIME RESISTANT; CEFEPIME RESISTANT; MEROPENEM RESISTANT

SUPPLEMENTAL ANTIBIOTICS

COLISTIN INTERMEDIATE; FOSFOMYCIN RESISTANT

Which antibiotic can be used for treatment purposes?

**ChatGPT’s Answer:**

The antibiotic resistance profile you provided indicates resistance to a broad range of antibiotics, including first-line, second-line, and supplemental antibiotics. However, there are limited options remaining. Given the resistance pattern, one potential option could be colistin. Colistin is a last-resort antibiotic and is often reserved for the treatment of multidrug-resistant infections.

It's important to note that the use of colistin is associated with potential toxicities and should be carefully considered by a healthcare professional. Additionally, consultation with an infectious disease specialist is advisable in such cases to ensure appropriate and effective treatment. The choice of antibiotic should be based on a thorough evaluation of the patient's condition, the specific bacteria causing the infection, and local resistance patterns.
